# Supplementary material for: Phase-selective recrystallization makes eutectic high-entropy alloys ultra-ductile
Source: Nat Commun. 2022 Aug 10;13:4697. doi: 10.1038/s41467-022-32444-4 (PMC9365806; doi:10.1038/s41467-022-32444-4)
Supplement: Supplementary file 1 — Supplementary Information [file 41467_2022_32444_MOESM1_ESM.pdf]

Supplementary information for

Phase-selective recrystallization makes eutectic high-entropy alloys ultra-ductile

Qingfeng Wu[1*], Feng He[1*], Junjie Li[1], Hyoung Seop Kim[2✉], Zhijun Wang[1✉], Jincheng Wang[1✉]

1-State Key Laboratory of Solidification Processing, Northwestern Polytechnical University, Xi'an 710072, China

2-Department of Materials Science and Engineering, Pohang University of Science and Technology, Pohang, Korea

* *These authors contributed equally: Qingfeng Wu, Feng He*

✉ *The corresponding authors: hskim@postech.ac.kr, zhjwang@nwpu.edu.cn, jchwang@nwpu.edu.cn*

This file includes:

Supplementary Figs. 1-13

Supplementary Tables 1 and 2

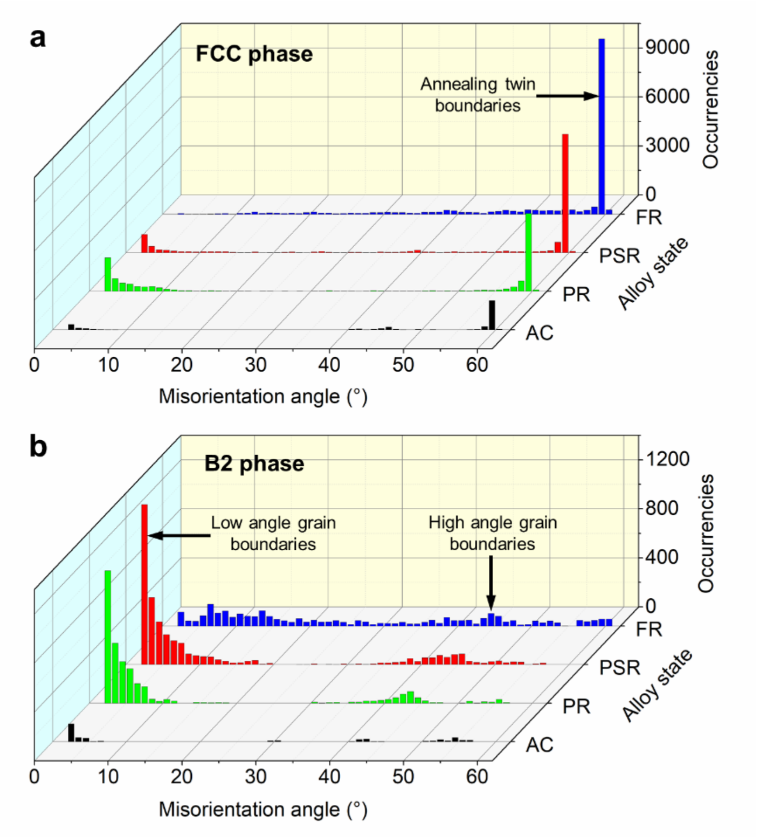

Supplementary Fig. 1. Evolution of the misorientation angle distribution during recrystallization. a, FCC phase. **b,** B2 phase. AC, PR, PSR, and FR represent ac-cast, partially recrystallized, phase-selectively recrystallized, and fully recrystallized EHEAs, respectively, as denoted in Methods. For the FCC phase, the density of twin boundary increases gradually in the AC, PR, and PSR EHEAs, indicating the occurrence of recrystallization. For the B2 phase, the density of low angle grain boundary increases while that of high angle grain boundary remains almost unchanged in the AC, PR, and PSR EHEAs, indicating the recovery rather than recrystallization. In the FR EHEA, the density of low angle grain boundary in both the FCC and B2 phases decreases significantly, indicating the full recrystallization of the two phases.

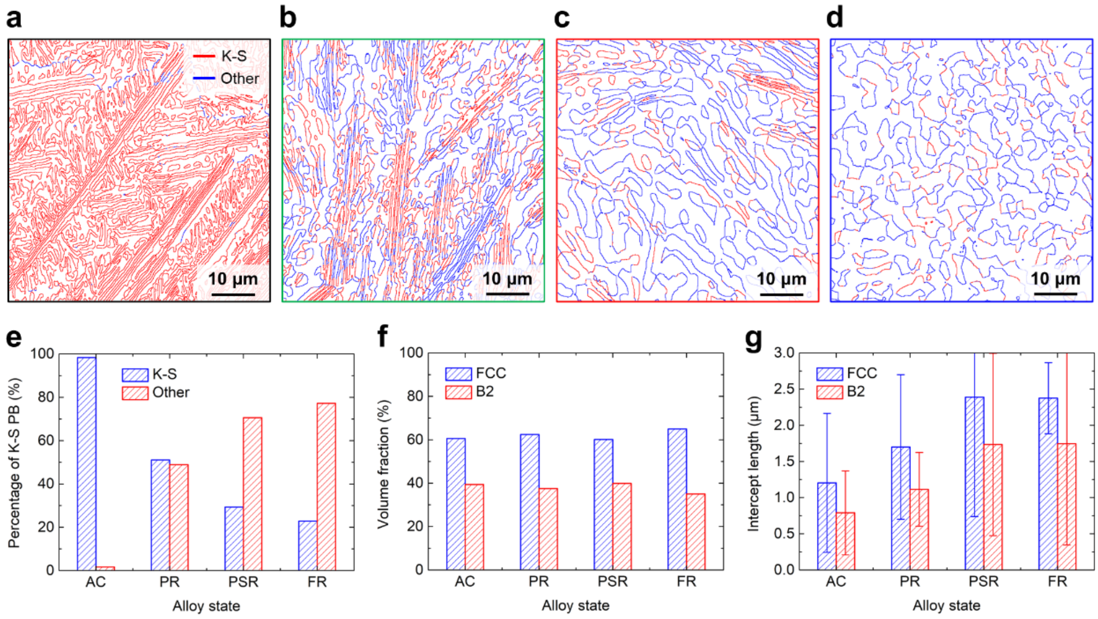

Supplementary Fig. 2. Evolution of the orientation relationship, phase volume fraction, and intercept length during recrystallization. **a-d,** Orientation relationship between the FCC and B2 phases in the AC, PR, PSR, and FR EHEAs, respectively. **e-g,** Statistical results of the percentage of Kurdjumove-Sachs (K–S) orientation relationship, intercept length and phase volume fraction, respectively. Error bars in **g** represent standard deviation. In the AC EHEA, the FCC and B2 phases share the K-S orientation relationship. With the increased content of recrystallization. the percentage of K-S interface decreases gradually, the volume fractions of FCC and B2 phases remain unchanged at ~60% and ~40%, while the intercept lengths increase gradually. Especially, the FR EHEA exhibits similar intercept length with the PSR EHEA, excluding the effect of phase sizes on mechanical properties.

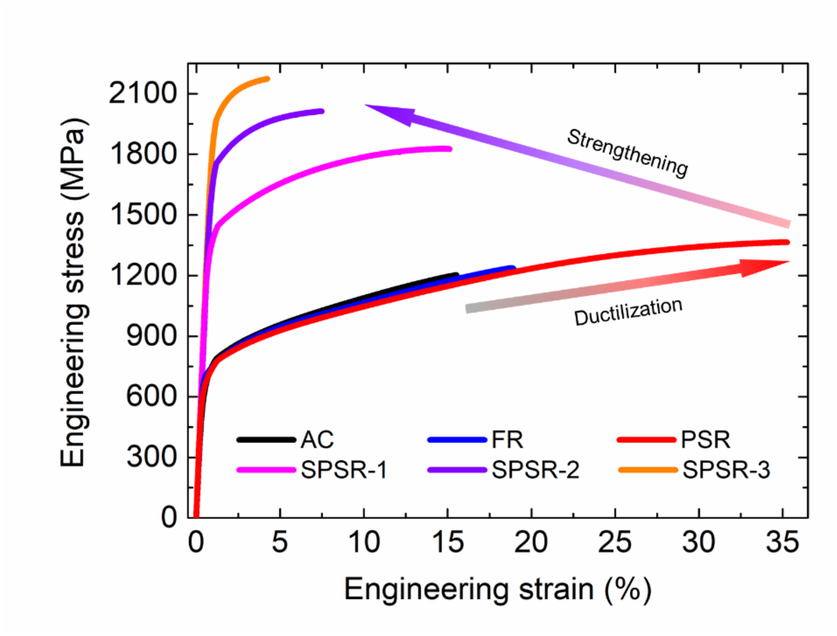

Supplementary Fig. 3. Tensile engineering stress-strain curves of the AC, FR, and PSR EHEAs and further strengthened PSR EHEAs. The AC and FR EHEAs exhibit similar tensile properties, with the tensile elongation of ~15% and ~19%, respectively. After PSR, the tensile elongation increases to ~35%, indicating a significant ductilization effect. After further strengthening by introducing dislocations and precipitations (denoted as the SPSR alloy, as shown in Methods), the ultimate tensile strength can be tuned from ~1.8 to ~2.2 GPa at the expense of ductility.

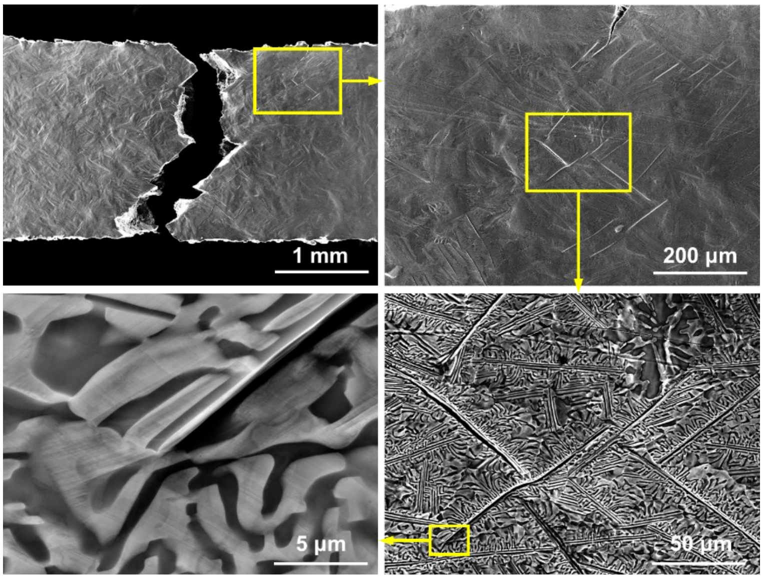

Supplementary Fig. 4. Cross-sections of the fractured AC EHEA. The main crack exhibits a zig-zag morphology. A large number of straight microcracks having an angle of ~45° to the tensile direction exist near the crack tip. High magnification images reveal the severe bending and slip deformation of the lamellar structures. Microcracks prefer to initiate at the phase boundaries in the lamellar regions.

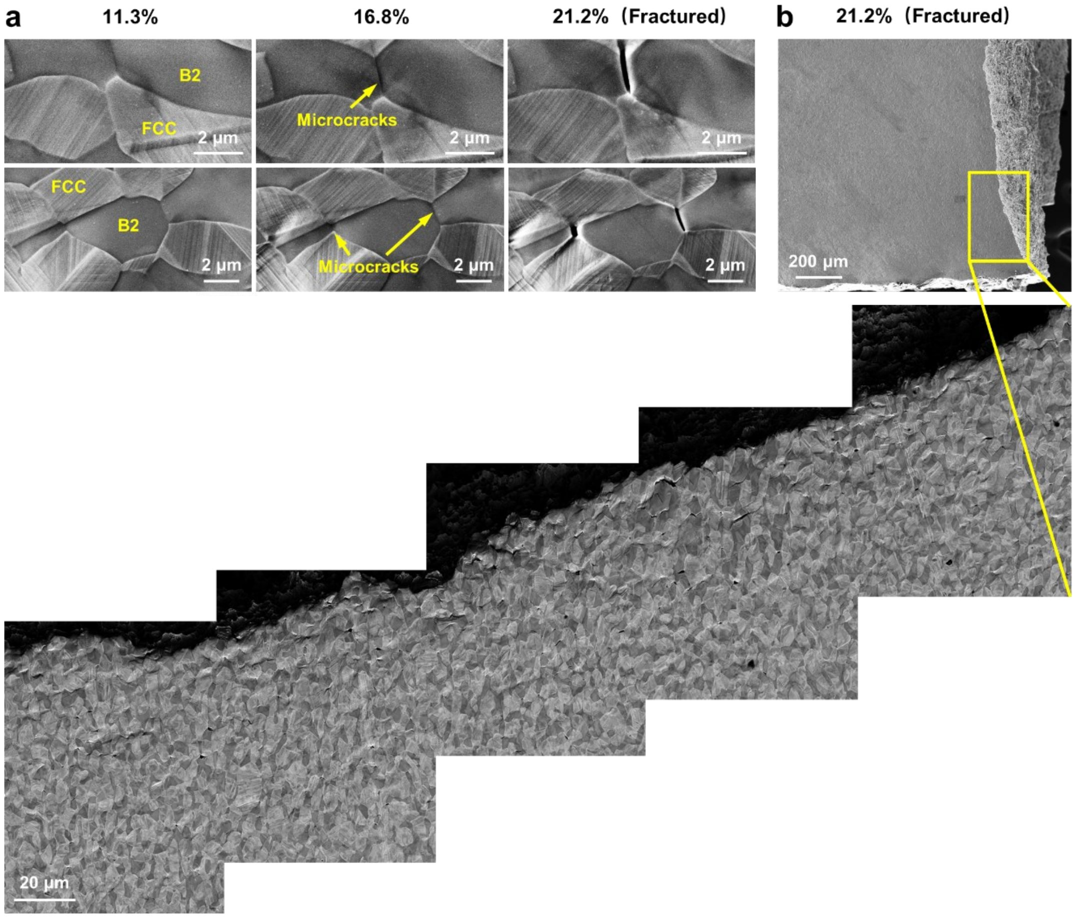

Supplementary Fig. 5. Specimen surfaces of the FR EHEA during in situ tensile tests. a, Formation and evolution of typical microcracks. We only observed microcracks inside the B2 phase, which appear at ~16.8% nominal strain. **b,** Macroscopic cross-section near the crack tip and corresponding high magnification images for measuring the microcrack density, as shown in the inset of Fig. 2c.

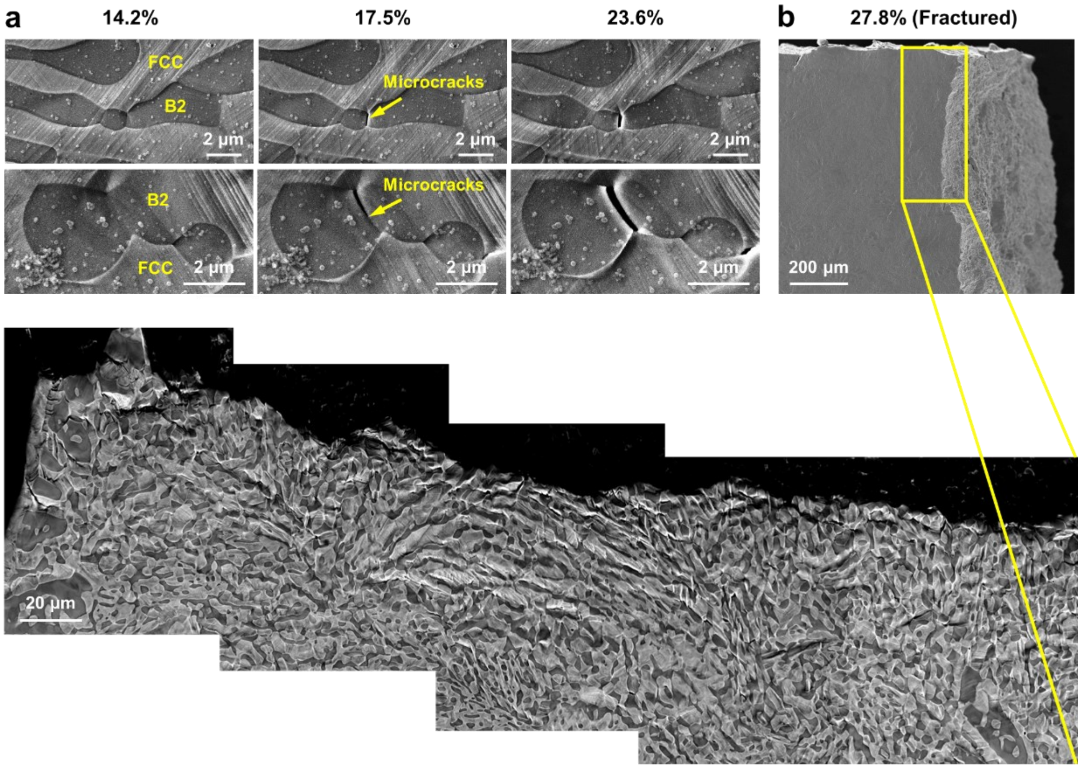

Supplementary Fig. 6. Specimen surfaces of the PSR EHEA during in situ tensile tests. a, Formation and evolution of typical microcracks. Similar to the FR EHEA, only microcracks inside the B2 phase are detected, which appear at ~17.5% nominal strain. **b,** Macroscopic cross-section near the crack tip and corresponding high magnification images for measuring the microcrack density, as shown in the inset of Fig. 2d.

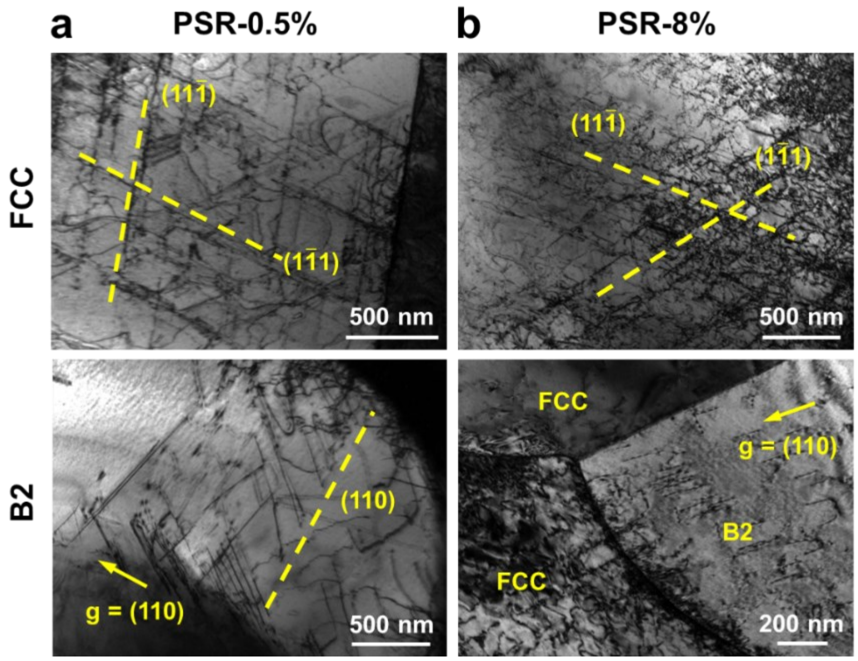

Supplementary Fig. 7. Dislocation substructures of the PSR EHEA at various nominal strains. a, 0.5%. **b,** 8%. After yielding (0.5% nominal strain), clear {111} and {110} slip traces are observed in the FCC and B2 phases, respectively, revealing the same planar slip mode. As the nominal strain increases to 8%, the dislocation density significantly increases in both the FCC and B2 phases, revealing the dislocation-dominated deformation mechanism.

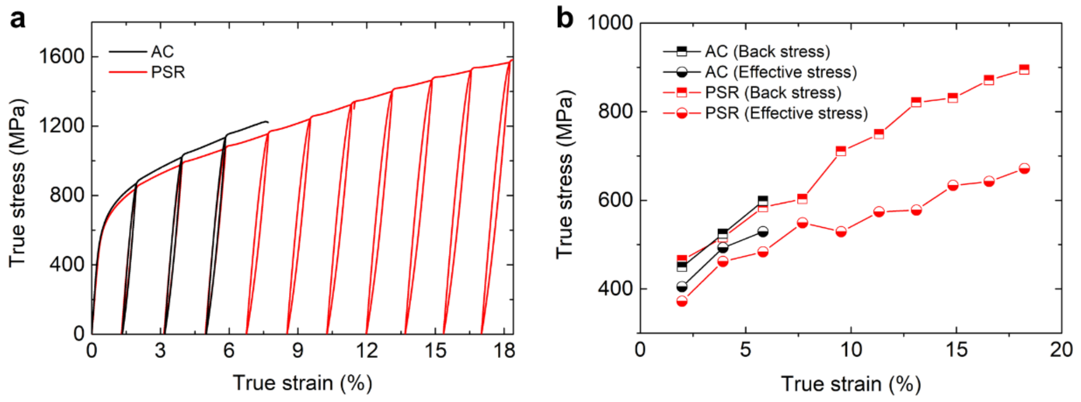

Supplementary Fig. 8. Comparison of the back stress of the AC and PSR EHEAs. **a,** Loading-unloading-reloading true stress-strain curves of the AC and PSR EHEAs. **b,** Variation of the back stress and effective stress with true strain in the AC and PSR EHEAs. Both the AC and PSR EHEAs exhibit obvious Bauschinger effect, indicating the effective hetero-deformation induced strengthening. The back stress of the two alloys is similar at the same nominal strain. Accompanied by the sustainable deformation, the back stress increases gradually, reaching up to ~900 MPa in the PSR EHEA near failure.

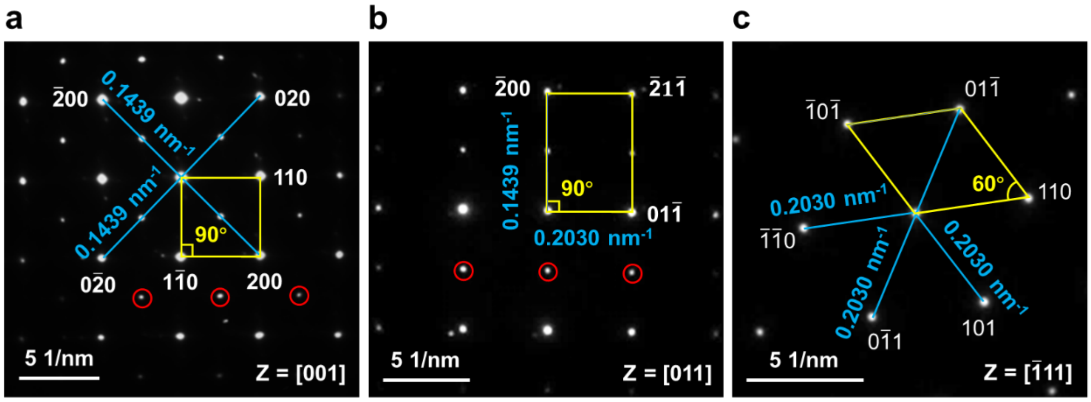

d

Crystal indices	Lattice spacing before deformation (nm)	Lattice spacing after deformation (nm)	Percentage change (%)
(200)	0.1439	0.1519	5.56
(020)	0.1439	0.1371	-4.73
(110)	0.2030	0.2024	0.00
(101)	0.2030	0.1888	-7.00
(011)	0.2030	0.2033	0.00

Supplementary Fig. 9. Comparison of the interplanar spacings of the B2 phase in the PSR EHEA before and after deformation. a-c, Selected area electron diffraction patterns of the B2 phase before deformation taken from the zone axis of [001], [011], [$\bar{1}$11], respectively. **d,** Comparison of specific interplanar spacings before and after deformation. Compared with the undeformed state, the interplanar spacing of (200) increases ~6%, while that of (020) decreases ~5%. Accordingly, the interplanar spacing of (101) decreases ~7%, while that of (110) and (011) remain unchanged. Therefore, we can confirm a B2 → body-centered tetragonal phase transformation achieved by expanding along the (200) direction and compressing along the (002) and (020) directions during deformation.

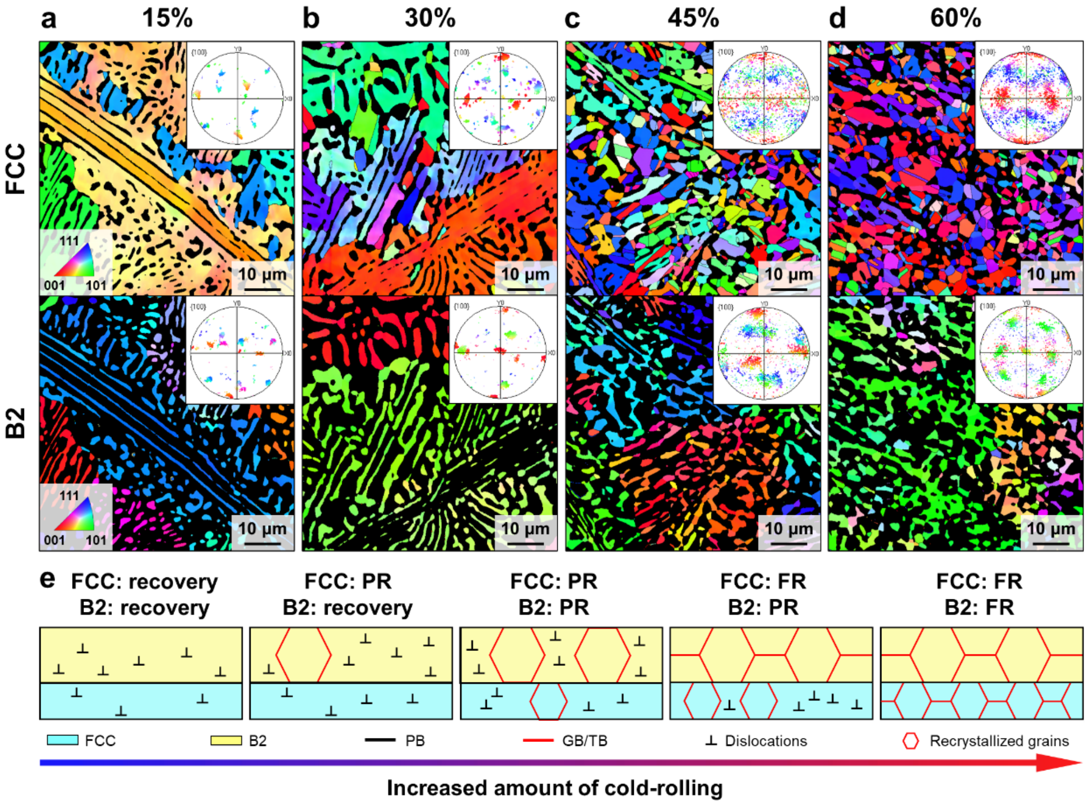

Supplementary Fig. 10. EBSD IPF maps of the FCC (upper row) and B2 (lower row) phases of the AC EHEA after rolling for different deformation amounts and annealed at 1200 °C for 20 mins. a, 15%. **b,** 30%. **c,** 45%. **d,** 60%. For the 15%-rolled and annealed alloy, neither the FCC nor B2 phases recrystallizes, but only recoveries. For the 30%-rolled and annealed alloy, the FCC phase recrystallizes partially, while the B2 phase recoveries. For the 45%-rolled and annealed alloy, the recrystallization volume fraction of FCC increases, and the B2 phase begins to recrystallize. For the 60%-rolled and annealed alloy, the FCC phase recrystallizes completely, while the B2 phase recrystallizes partially. **e,** Schematic showing the recovery and recrystallization behaviors of the FCC and B2 phases under different deformation amounts and subsequent annealing.

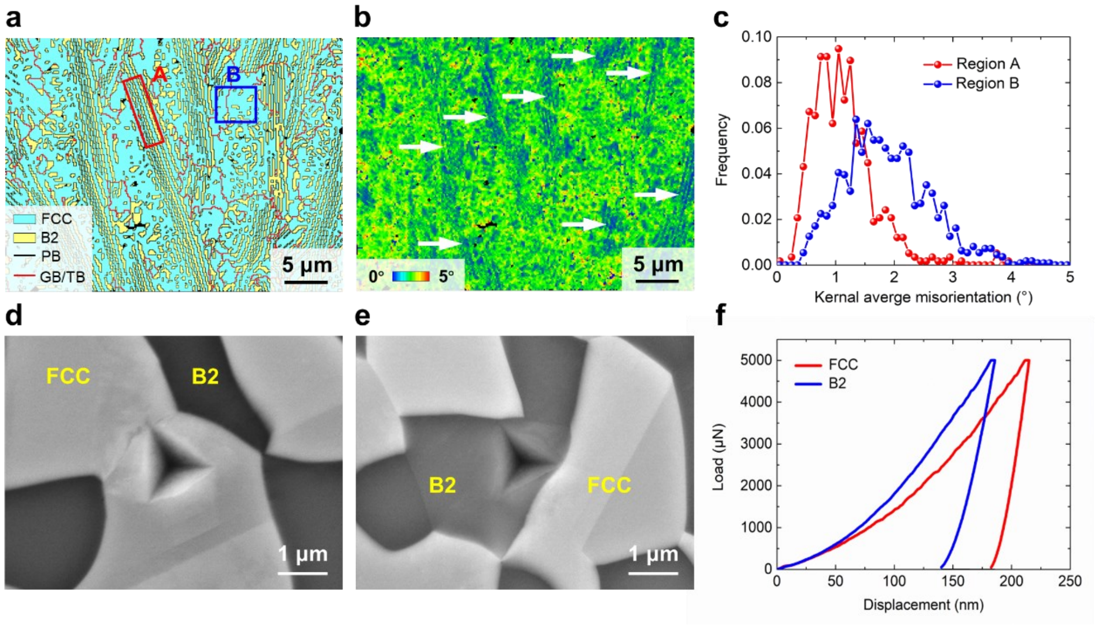

Supplementary Fig. 11. Mechanisms for the preferential recrystallization of irregular region and FCC phase. a and b, EBSD phase and Kernel average misorientation (KAM) maps of the cold-rolled AC EHEA, revealing the much lower lattice rotation in the lamellar regions than in the irregular regions, as indicated by the white arrows. **c,** Statistical results of the KAM value in the A and B regions marked in **b**. The severe lattice rotation in the irregular regions is responsible for the preferential recrystallization in the PR EHEA. **d and e,** SEM images confirming the phase-specific indentations of FCC and B2 in the PSR EHEA. **f,** Load-displacement curves for the FCC and B2 phases. The FCC phase shows a lower microhardness of ~4.44 GPa than the B2 phase of ~5.67 GPa, making it easier to deform during deformation and accumulate more strain energy for recrystallization.

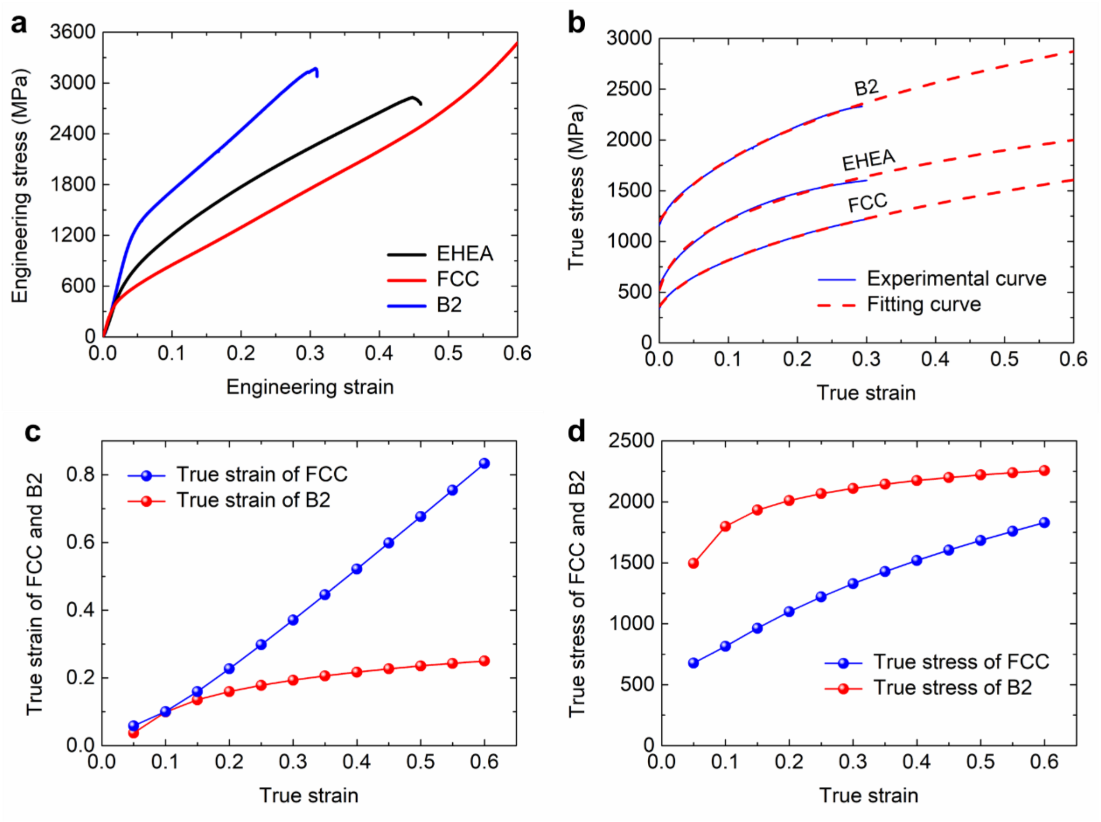

Supplementary Fig. 12. Estimation of the strain and stress partitioning in the FCC and B2 phases at different global strains. a, Compressive engineering stress-strain curves of the FCC, B2, and EHEA alloys. **b,** Experimental true stress-strain curves of the FCC, B2, and EHEA alloys with only the plastic strain of 0-30% considered and corresponding fitting curves. **c,** Calculated average true strain in the FCC and B2 phases at different global strains. **d,** Calculated average true stress in the FCC and B2 phases at different global strains.

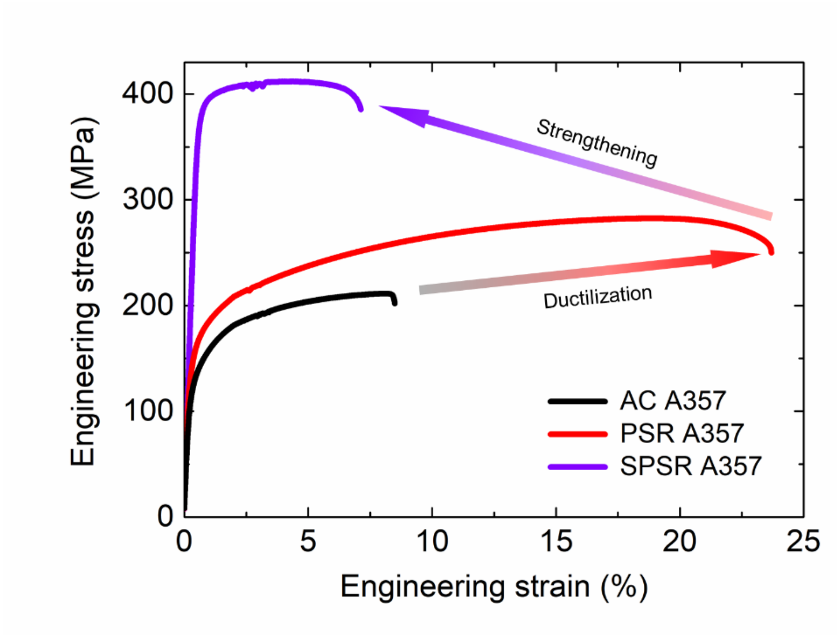

Supplementary Fig. 13. Tensile engineering stress-strain curves of the AC, PSR and further strengthened PSR A357 casting aluminum alloys. The AC A357 alloy exhibits poor tensile elongation of ~8%. After PSR, the elongation increases to ~23%. After further strengthening by introducing dislocations and precipitations, the SPSR A357 alloy exhibits a high tensile strength of ~400 MPa, double that of the as-cast state. These results prove the universality of PSR and further strengthening routes for various eutectic systems.

Supplementary Table 1. Fitted parameters of K_i, $\varepsilon_{0,i}$, and n_i (i=E, FCC, B2) in Supplementary Fig. 12b.

Alloy	K_i, MPa	$\varepsilon_{0,i}$	n_i
EHEA	2311.81 ± 1.36	0.0059 ± 0.000076	0.2894 ± 0.000388
FCC	1956.46 ± 0.43	0.0157 ± 0.000039	0.4058 ± 0.000178
B2	3294.30 ± 1.47	0.0358 ± 0.000170	0.3023 ± 0.000435

Supplementary Table 2. Calculated strain in the FCC and B2 phases at different global strains (%).

Global engineering strain	Global true strain	Average true strain in FCC	Average engineering strain in FCC	Average true strain in B2	Average engineering strain in B2
15	16	17	16	15	14
30	36	46	37	21	19
45	60	83	56	25	22
